# Type 2 Innate Lymphoid Cell (Ilc2)-Deficient Mice Are Transcriptionally Constrained During *Nippostrongylus brasiliensis* Infection

**DOI:** 10.3390/pathogens14060571

**Published:** 2025-06-07

**Authors:** Damarius S. Fleming, Fang Liu, Joseph F. Urban, Robert W. Li

**Affiliations:** 1 USDA-ARS, Beltsville Agricultural Research Center, Animal Parasitic Diseases Laboratory, Beltsville, MD 20705, USA; 2 College of Public Health, Zhengzhou University, Zhengzhou 450001, China; fliu19910205@gmail.com

**Keywords:** gastrointestinal, gene expression, Ilc2, immune response, helminth

## Abstract

Mouse models serve as a means of examining immune changes when genes of interest are knocked out (KO). One group of immune gene-producing cells that have been identified is type 2 innate lymphoid cells (Ilc2). These cells are involved in the production of Th2 equivalent immune responses and signal cytokine production during the resolution of *Nippostrongylus brasiliensis* parasite infection in mice lungs. However, many questions about Ilc2 activity in the gut remain. To study this, retinoic acid receptor (RAR)-related orphan receptor alpha (*RORα*)-deficient mice were infected with adult *N. brasiliensis* and arranged into four treatment groups. Ten days post-infection (dpi), mouse ileum tissue was extracted for RNA-Seq. The *RORα*-deficient mice showed little change in gene expression at 10 dpi (N = 51) when compared to the WT mice at 10 dpi (N = 915), displaying dysregulation within the mouse gut. Based on the results, the gene expression in the gut of Ilc2-deficient mice denoted that the inability to craft Ilc2 cells left the mice unable to mount classical helminth immune responses involving humoral, mast cell, and antibody Th2-driven reactions. Overall, the results showed the importance of Ilc2 in the gut during *N. brasiliensis* infections and the effect that the lack of these cells had on immunity.

## 1. Introduction

The ability of research to improve human and livestock health has been strengthened using mouse models that act as proxies for host–pathogen interactions. Mouse models serve as an efficient means of examining immune and metabolic changes at the genome level and investigating the consequences of turning off, or knocking out (KO), genes of interest. Mice infected with *Nippostrongylus brasiliensis*, a mouse hookworm, model human immunogenetic responses to hookworm infections [1,2]. The information gained from these mouse models is also informative for other helminth infections seen in livestock settings, especially in the case of immune response cells. One group of immune cells that has been identified, i.e., type 2 innate lymphoid cells (Ilc2), promotes the production of Th2 equivalent immune responses that sync with adaptive immunity and signal cytokines during the clearing of *N. brasiliensis* infections in mice [2,3]. Ilc2 cell production initiates cytokine and eosinophilic responses to helminth parasites in immune cells situated within the epithelial barriers of the lung and gut. Although prior studies have characterized their activity in mouse lung tissue, less is known about the function of Ilc2 cells in the mucosal linings of the gut [1,3,4]. This is especially true when considering the immunogenetic impact that a lack of Ilc2-mediated immune responses has on host–parasite interactions. The role of Ilc2 cells in mediating host–parasite interactions deserves more attention. The retinoic acid receptor (RAR)-related orphan receptor alpha (*RORα*) is a necessary precursor of type 2 innate lymphoid cells (Ilc2) [5,6] and has roles in host metabolism and inflammation. Previous studies have shown that *RORα*-deficient mice suffer from higher *N. brasiliensis* worm burdens [7]. The lack of *RORα*, which is critical to the stimulation of Ilc2, affords KO mice less protection at mucosal sites within the gut. For helminth infections, Ilc2 proliferation at sites within the gastrointestinal (GI) environment is a key portion of the host immune response [8]. Therefore, we endeavored to elucidate the transcriptional consequences and resulting immunogenetic effects of disrupting Ilc2 function in the mouse gut during *N. brasiliensis* infections.

## 2. Materials and Methods

### 2.1. Animals

We employed knock-out (KO) mice deficient in the related orphan receptor alpha (*RORα*), which is needed for downstream production of Ilc2. We prepared these animals according to the protocols described in Guo et al., 2015 [7]. We challenged the mice (day 0) subcutaneously using a bolus of 500 L3 *Nippostrongylus brasiliensis* (Nb) larvae, strain MIMR, inoculated in the inguinal region of the mice. The mice used were wild-type C57BL/6J and Jax line 2651 in the B6 background (RORa^KO/+^) (aged approximately 10 days), which were ordered from the Jackson Laboratory (Bar Harbor, ME, USA) and used as donors. Receipt line *Rag2*^−/−^*Il2rg*^−/−^ mice at 6 to 10 weeks of age (Line 4111) were ordered from Taconic Biosciences (Germantown, NY, USA. The mice were group-housed (4 mice per cage) and had free access to water and a standard chow diet. All the animal experiments were performed under approval of the Animal Care and Use Committee of the National Institute of Allergy and Infectious Diseases (US National Institute of Health). A total of 13 mice were used to create the four treatment groups (KO_NB_10dpi, WT_NB_10dpi, KO_control, and WT_control) examined in this study. Day 10 post-infection was used to observe the later larval and adult stages of the infection as it went from the lung to the intestine.

### 2.2. RNA Samples

Host total RNA was extracted from the control and KO mice ileum tissue 10 days post-infection using a Qiagen RNeasy Micro Kit (Germantown, MD, USA). Library construction and sequencing were performed in-house at the USDA animal parasite unit (APU) on an Illumina NextSeq (™) (San Diego, CA, USA) instrument, which produced 13 total single-end reads for gene expression analysis. An outlier for the KO mice was detected and removed based on a principal component analysis (PCA) test that showed a KO control sample grouped outside the KO_NB_10dpi samples and the WT control samples. This sample from the KO control group was removed before expression analysis, leaving a total of 12 samples (3 reps per treatment group) for the final analysis.

### 2.3. RNA Analysis

Quality control of the single-end reads for each treatment was performed using FastQC and MultiQC [9,10], with no trimming of the reads; only reads with a quality score ≥ 25 were retained for downstream analysis. The reads were mapped to the current mouse genome and GTF file, GRCm39, using HISAT2 [11] with default scoring and alignment options. The BAM files were run through StringTie, along with the current mouse GTF file for transcript assembly and splice variant quantification. The output from StringTie and the mouse GTF was converted to a GFF3 file. Only those transcripts that could be matched to the Ensembl GTF were retained and used to annotate the raw count data. Raw gene counts were calculated using FeatureCounts [12,13]. The arguments within FeatureCounts were set to filter out reads with a quality score > 25 and to count split and non-split alignments, while prohibiting read multi-mapping prior to gene expression calculations using DESeq2 [14]. Pre-filtering of the count matrix removed any gene that had a row total of zero prior to fold-change analysis within DESeq2. Parameters for DESeq2 were set to default values for the *estimateSizeFactors* argument; *Fit type* was set to “local”. Additionally, the options for outlier and independent filtering were set to “yes.” The final gene lists were subjected to an FDR of ≤0.05. The treatment groups producing extremely large gene lists were subjected to a second filter for fold change > 2 (log2FC) to facilitate gene ontology and pathway analysis.

### 2.4. Gene List Function and Pathway Analysis

The gene expression lists from each group were further explored using the software gProfiler (version e113_eg59_p19_6eaea45) and STRINGdb Ver. 12.0 set to use all known genes annotated to *Mus musculus* to allow for the highest number of query genes to be recognized [15,16]. Further information regarding pathways and gene effects was examined using Ensembl, Uniprot, and NCBI [12,17,18]. An adjusted *p*-value (FDR) of 0.05 was used for significance.

## 3. Results

### 3.1. Gene Expression, Gene Ontology, and Pathway Analysis

Gene expression analysis of the four treatment group comparisons was used to examine the differences in host immune responses of the wild-type (WT) and the RORα knock-out (KO) mice at 10 days post-infection with *N. brasiliensis*. Comparison of these groups enabled the assessment of the transcriptional implications of Ilc2 deficiency during parasitic infection. We started by examining Ilc2 deficiency in the absence of infection by comparing the uninfected WT mice to the uninfected KO mice. We then contrasted the reactions of the WT and KO groups after 10 days of infection.

### 3.2. WT_Control vs. KO_Control

The comparison of the two control groups produced only 22 differentially expressed genes (DEGs) meeting the significance threshold. Of the 22 genes, only 3 (14%) were upregulated in the KO control; the remaining 19 (86%) were downregulated (Table 1). Many of the DEGs were immunoglobulin heavy or kappa variable molecules, such as immunoglobulin heavy variable 1-82 (Ighv1-82) and immunoglobulin kappa variable 5-48 (Igkv5-48). The immunoglobulin genes were annotated to be involved in the adaptive immune process and part of the secretory complexes for IgA, IgG, and IgM [17,18]. The immunoglobulin genes were joined by other downregulated genes related to the initiation of the complement system and antigen presentation to the mouse MHC II. The upregulated genes were only predicted genes and pseudogenes lacking annotation. Consequently, the only annotated genes undergoing differential expression were downregulated in the KO mice. Together, these DEGs promote antigen binding, phagocytosis, and adaptive immune response initiation of B and T cells [17,18].

**Figure 1:**
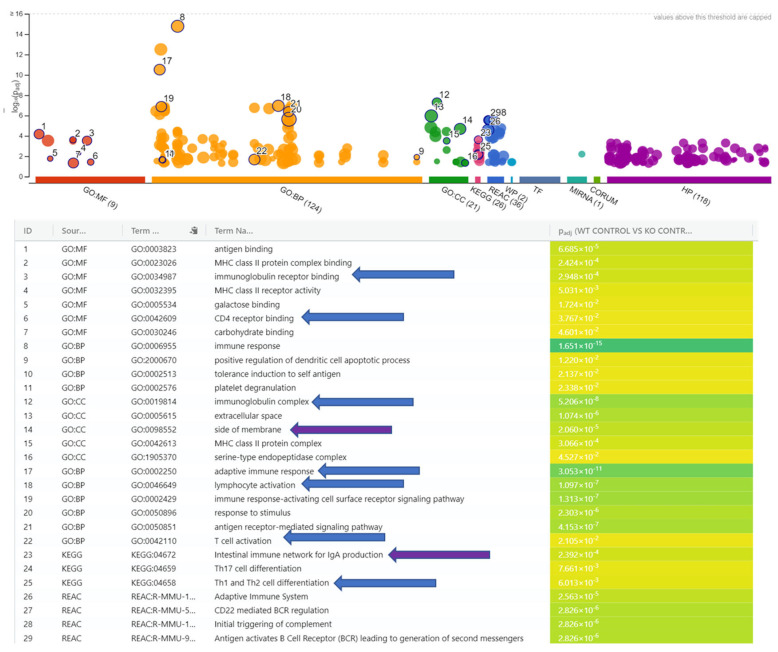
Pathways impacted by non-functioning Ilc2 in the RORα knock-out (KO) control vs. wild-type (WT) control mice. Compared to the wild-type mice, the inability of the KO mice to transcribe Ilc2 causes a defect in immunoglobulin complex genes that predisposes them to downregulated immune processes. The most notable, including lymphocyte activation, immunoglobulin receptor binding, CD4 receptor binding, Th1 and Th2 cell differentiation, complement and adaptive immune response activation (blue arrows), indicated where the issues would take place: the side of the membrane and the intestinal immune network for IgA production (purple arrows).

The short list was run through gProfiler (version e113_eg59_p19_6eaea45) (Figure 1) and STRINGdb (Ver. 12.0) to ascertain any pathways affected by their downregulation. The threshold for significance was set to an FDR of 0.05 and utilized all known genes from the current mouse genome build. Each analysis highlighted pathways implying the KO group’s inability to properly trigger downstream immune processes, including the complement system, adaptive immunity, and B and T cell activation, and pointed out genes related to abnormal gastrointestinal and immune physiology. Additionally, all the pathways recognized were downregulated in the KO mice because the gene list was comprised of only downregulated genes. The most prominent genes regarding pathways appeared to be CD79A antigen (immunoglobulin-associated alpha), IGHV1-5, IGHV1-53, IGHV1-82, IGKV1-117, and tumor necrosis factor receptor superfamily member 13b (TNFRSF13B). There were two DEGs with direct lymphocyte and T cell connections: B lymphoid kinase (Blk) and Lymphocyte transmembrane adapter 1 (Lax1). The gene Blk is involved in B-lymphocyte development, but it also acts to transmit signals to immunoglobulins. It is also a negative regulator of leukocyte proliferation, so its downregulation could possibly increase the functions of the innate immune response [12,17,18,19].

### 3.3. WT_Control vs. WT_NB_10dpi

We next examined the transcriptional responses of the wild-type mice to infection by comparing the infected and uninfected mice with functional Ilc2 cells. Infection induced differential expression of thousands of genes. Specifically, 5496 genes passed our false detection threshold; the majority of these, amounting to 3003 (55%), were downregulated in the WT_NB_10dpi mice. Because of the large number of DEGs, we focused on those genes that underwent a log2 fold change (fc) of 2 or greater. This reduced the list to N = 915 with 279; of these, 636 (70%) were downregulated in the WT_NB_10dpi mice (Supplementary Table S1). Among this large number of DEGs, a further comparison to the KO mice (below) drew particular attention to 23 genes of elevated interest. These genes included five that were identical between the WT and KO at 10 dpi; the other 18 genes are paralogs of each other (Table 2).

We examined the pathways most affected by the differentially expressed genes, as a consequence of infection, in the WT mice benefiting from functional Ilc2 cells at 10 dpi. Doing so revealed key parasite immune response enrichments (Table 3) in biological processes, such as antimicrobial humoral immune response mediated by antimicrobial peptide (GO:0061844), which included 14 upregulated genes, and alpha-defensins (MMU-1462054), including 12 upregulated defensin genes that appear in multiple pathways. In total, the defensin genes made up a large portion of other immune-related pathways as part of the humoral response. Another gene group contributing to multiple gene networks included regenerating islet-derived gene homologs that clustered around functional terms related to helminth infections, such as cell wall disruption in other organism and Cobalamin (Cbl, vitamin B12) transport and metabolism (CL:25609), mixed, incl. activation of matrix metalloproteinases, and cell wall disruption in other organism (CL:25564). Also of interest was the over-enrichment of genes expressed in the tissues of the WT_NB_10dpi mice. Other tissues with enriched gene expression in the WT_NB_10dpi mice included the muscular system (BTO:0001485). The genes that mapped to the muscular system were all downregulated at 10 dpi, which may denote genes related to muscle action expressed for parasite expulsion. The pancreas- and ileum-related DEGs (n = 26) reinforced the need for digestive enzymes and glucagon to power the return to homeostasis in response to helminth infection. The most telling term to come out of the tissue expression terms was M2 macrophage (BTO:0006111), including arginase (ARG1), which is a negative regulator of T cell activity and the type II interferon-mediated signaling pathway that contributes to protection by promoting Th2 responses in the mouse lung [20]. Lastly, there was chitinase-like 3 (CHIL3), also known as YM1, which is thought to function as a pro-inflammatory gene and has also been shown to be protective against lung injury in parasite infections [21]. Overall, the WT mice displayed differential regulation of various pathways, underscoring immune and physiological responses for clearing the parasite, performing repairs, and moving back towards homeostasis.

### 3.4. KO_Control vs. KO_ NB_10dpi

We next examined the immune responses to infection in the mice lacking functional Ilc2, comparing them to the uninfected mice also lacking in Ilc2. This allowed us to distinguish the immunological consequences of Ilc2 deficiency from any broader physiological changes resulting from the knockout condition. In contrast to the mice with functional Ilc2 (where infection induced differential expression in over 5000 genes), infection induced differential expression in only 51 genes in the mice lacking functional Ilc2. Of these, 29 (57%) were upregulated in the infected mice. Evidently, a broad array of immunological responses to infection with *N. brasiliensis* require functional Ilc2. Some of the downregulated genes of interest appear to point to the KO mice not being able to control parasite blood feeding and tissue damage or adjust inflammatory responses caused by infection. The most curious of the downregulated expressed genes included MicroRNA 196a-1, which itself has been shown to target the expression of collagen 1 and α-smooth muscle actin, which can cause kidney damage [22]. Also, the gene ATP-binding cassette, subfamily A, member 12 (Abca12), was downregulated in the 10 dpi infected mice, which has been characterized in dermatological studies to be essential to skin barrier repair and may show a lack of this ability without functional Ilc2 [23]. The gene UL16 binding protein 1 (Ulbp1) is characterized as an upstream immune process effector with the ability to positively regulate interferon and leukocyte activation. It is thought to directly regulate the receptor binding action of natural killer cell lectin-like, which had a paralog that appeared in the KO results as an upregulated immune response gene [12,17,18]. Although not well characterized as a gene of immune importance to *N. brasiliensis* infections, Ulbp1 appears to be an important part of biological processes linked to Ilc2 competent immune activity. The gene is a ligand for killer cell lectin-like receptor subfamily K, member 1 (Klrk1), also called Nkg2d, which is crucial to MHC activity within the GI tract and lung, as well as other key immune responses and disease progressions [17,24,25]. The most downregulated gene was Solute carrier family 40 (iron-regulated transporter), member 1(Slc40a1), which is involved in iron transfer from the mouse duodenal epithelium. The lowered expression of this gene may be an indication that the KO mice were still experiencing nutritional and physiological deficits, hindering iron and lymphocyte homeostasis. The results showed slight upregulation of some immune genes, some of which elicit a cytokine response in the absence of Ilc2. One of these genes, killer cell lectin-like receptor subfamily H, member 1 (Klrh1), is a cell surface pattern receptor that acts to bind antigens. It also functions as a positive regulator of a host of immune processes that include lymphocyte activation and cytokine production involved in the inflammatory response [17,18]. There was also the gene insulin receptor substrate 2 (Irs2), which can mediate cytokine responses [17,18].

Most of the genes upregulated in the KO mice seemed to involve metabolic rather than immune responses. Other upregulated genes of interest included glutathione S-transferase, mu 3 (Gstm3), which assists in the cellular response to xenobiotic stimulus and thus may have resulted from the parasites actions of invasion or utilization of host resources [2]; liver-expressed antimicrobial peptide 2 (Leap2), an active part of the humoral immune response and the only upregulated antimicrobial gene in the list; and Tribbles pseudokinase 3, Tribbles homolog 3 (Trib3), which interacts with multiple MAPK immune pathways, autophagy, and apoptosis [17,18]. The higher expression at 10 dpi may be related to the presence of worms or eggs in the ileum, serving as a constituently active alarm for the “stalled” host innate response with no way to initiate a proper Th2 response. It has been shown that RORα-deficient mice are not able to efficiently expel adult worms and carry high parasite egg burdens when compared to wild-type mice. Additionally, there were genes shared with the WT mice infected at 10 dpi that may shed more light on how the immune response of the KO mice is transcriptionally altered by the exclusion of Ilc2.

## 4. Discussion

### 4.1. Mice Without Ilc2 Experience Downregulated Immunoglobulin Complex Immune Response and Transcriptional Regulators to N. brasiliensis Infection

The lack of a transcriptional initiator for Ilc2 handicaps responses to *N. brasiliensis* at 10 dpi. The wild-type mice (with functional Ilc2) responded to infection by differentially expressing over 5000 genes, but only 51 genes underwent differential expression, as a consequence of *N. brasiliensis* infection, in the isogenic KO mice lacking functional Ilc2. Thus, Ilc2-generated immune molecules exert a powerful effect on the host’s response, influencing immunity, metabolism, and tissue repair. Examination of the uninfected controls showed that the mice lacking these specialized innate cells mounted a highly attenuated adaptive immune response to the parasite.

Infection induced most of the observed differences between the Ilc2-competent and Ilc2-deficient mice. Comparing such mice in the absence of infection identified only 22 experiencing differential expression; half of these were immunoglobulin genes downregulated in the KO mice. Most of these 11 genes are upstream actors of the complement and adaptive immune response. These genes are located in the plasma membrane, where they would have contacted the parasites.

This set of downregulated genes (Table 1) suggests that Ilc2 is needed for the host immune system to properly recognize and attack the parasite eggs deposited in the host GI. Most of the genes belong to IgA, IgG, and IgM complexes, causing downregulation of immune recognition; this deficient restructuring of immunoglobulin complexes reduces the diversity of antibody responses. The immunoglobulin gene results reinforce the commonality observed between Ilc2 and Th2 cells. The inability of the KO mice to combat the parasite was further supported by the downregulation of several homeobox (Hox) genes at 10 dpi. The downregulation of Hox genes Hoxb5, Hoxb7, and Hoxb8 seems to take part in modulating transcription. The gene Hoxb8 is a negative transcriptional regulator, but it also involves sensory perception of pain. The lowered expression in the KO mice could be a means of increasing the transcription of needed genes or a means of easing the dysbiosis generated by the parasite. Some of the downregulated genes of interest point to the inability of the KO mice to control parasite blood feeding, tissue damage, or adjust inflammatory responses during the parasitic infection. It is likely that examination of an earlier timepoint would show more dysregulation of the host immune system in the infected KO mice. However, it is possible that in the RORα-deficient mice, another regulation pathway, other than Ilc2, was damaged, preventing a proper inflammatory or immune regulation response from being carried out in the KO mice. It must be kept in mind that RORα deficiency disrupts multiple other biological functions, not just Ilc2 stimulation. However, it is probable that RORα deficiency is the principal cause for the lack of a proper Ilc2 response to the parasitic infection. The role it plays in macrophage actions may have altered the intensity and response of the immune system to the parasite. Macrophage activation is also an action of Ilc2, with the interaction during parasite infections being dependent on the interplay between M2 macrophages and Ilc2.

### 4.2. RORα-Deficient Mice Show Differential Expression of Paralogs of Immune-Related Genes Expressed in Wild-Type Mice Infected with N. brasiliensis at 10 Dpi

Infection induced differential expression in 23 genes, irrespective of the functionality of Ilc2. Of these, 6 were identical in KO and WT mice, and 17 were paralogs (Table 2). The six identical genes were all downregulated in the KO group at 10dpi, but they were highly upregulated in the WT mice. The results from this comparison spotlight gene candidates for vaccines or treatments to expel parasites and eggs because these represent key differences in Ilc2-dependent host responses to infection. The opposite direction of the expression of most of these genes (Table 2) underscores the need for functional Ilc2 in supporting immune and repair responses in the gut. For instance, the results showed the downregulation of some key immune genes affecting the immune response at 10 dpi. One such gene group was regenerating islet-derived 3 gamma (Reg3g), a C-type lectin that exhibits antimicrobial and wound healing properties but can also lead to inflammation in the mouse ileum, along with its paralog regenerating islet-derived 3 beta (Reg3b), which is involved in the inflammatory response and is a negative regulator of the inflammatory response to wounding [12,17,18]. Although changes in the regulation of inflammatory responses could be related to the lack of RORα, they appear to be predominantly related to a lack of Ilc2 expression. While both genes were only slightly downregulated in the KO group, they were strongly upregulated in the WT mice. The importance of the Reg3 genes is echoed in the results from WT_NB_10dpi, which presented over-enrichment of these upregulated genes clustered together under molecular functions that included the activation of matrix metalloproteinases and cell wall disruption in other organisms (Table 3). It appears that the WT mice also seemed to employ more Reg3 genes (Reg3a, Reg3d) as part of their immune response using antimicrobial peptides in the mouse gut.

Functional Ilc2 also influenced the intensity of differential gene expression in response to *N. brasiliensis* infection. These paralogs form connections to each other and with the four REG3 genes. Immunologically successful WT mice undergo strong upregulation of Ly6/Plaur domain containing 8 (Lypd8) and LY6/PLAUR domain containing 8 like (lypd8l), the former of which has exhibited anti-inflammatory properties in mucosal barriers [26]. The protective barrier function is likely the reason for its upregulation in response to infection by *N. brasiliensis*. This likely resulted from the genes being heavily expressed in the mouse intestine and playing roles in the host defense response to bacteria [12,17,18]. By contrast, infection induced only a slightly increased expression of Ly6/Plaur domain containing 1 (Lypd1) in the KO mice lacking functional Ilc2. This gene functions in acetylcholine receptor binding and inhibition and is a negative regulator of protein localization within the plasma membrane. Although part of the Ly6 protein family that can act as surface markers for immune targeting by antibody-mediated expression, *Lypd1* acts as a neurotransmitter and has not been recorded as a signal or marker for parasite targeting within the mouse GI. Other gene paralogs that underwent parallel changes in expression in the infected WT and KO mice included downregulated sperm acrosome-associated 6 (Spaca6) in the WT mice and sperm acrosome-associated 4 (*Spaca4*) in the KO mice. Perhaps infection diverted energy from reproduction to healing in each type of mouse. However, the paralog Spaca4 in the KO mice forms a connection with Ly6 genes and acts as a cell adhesion gene. In the end, the mice without Ilc2 expressed alternate immune response genes at 10 dpi. When compared against the wild-type mice, the KO mice at 10 dpi showed very little differential expression, characteristic of a strong immune or repair response. The few genes induced by infection in the Ilc2-deficient mice indicate some engagement of alternate pathways to re-establish homeostasis.

## 5. Conclusions

Although RORα deficiency in mice can alter multiple biological and immune functions, anti-parasitic functions linked to an inability to activate Ilc2 activation may be detrimental to parasite defense, expulsion, and gastric repair. The data from our study underscore that functional Ilc2 cell activation is key to a myriad of mouse responses to *N. brasiliensis* infection. These cells underpin classical helminth immune responses involving humoral, mast cell, and antibody Th2-driven reactions. It is likely that the examination of an earlier timepoint would show more dysregulation by the parasite of the host, driven by the lack of Ilc2 within the immune system. Overall, the results gave insight into the important role of Ilc2 in the gut during *N. brasiliensis* infections and the effect that the lack of these cells had on infections.

## Figures and Tables

**Table 1:** List of DEGs from the ILC2 WT control vs. the ILC2 KO control. The small list of genes suggests that the inability to produce ILC2 cells limited the ability of the mice to activate a proper immune response.

Ensembl_ID	log2(FC)	P-adj	Gene Name
ENSMUSG00000111709	2.49	7.62 × 10^−11^	Gm3776
ENSMUSG00000110275	2.36	1.70 × 10^−9^	Gm5905
ENSMUSG00000074196	1.46	1.49 × 10^−2^	Clca4c-ps
ENSMUSG00000010142	−1.19	2.38 × 10^−2^	Tnfrsf13b
ENSMUSG00000067341	−1.34	2.93 × 10^−2^	H2-Eb2
ENSMUSG00000041538	−1.37	2.01 × 10^−2^	H2-Ob
ENSMUSG00000051998	−1.38	1.51 × 10^−3^	Lax1
ENSMUSG00000093894	−1.39	4.95 × 10^−2^	Ighv1-53
ENSMUSG00000076939	−1.39	4.58 × 10^−2^	Iglv3
ENSMUSG00000014453	−1.45	2.01 × 10^−2^	Blk
ENSMUSG00000024863	−1.49	2.39 × 10^−2^	Mbl2
ENSMUSG00000026616	−1.50	2.01 × 10^−2^	Cr2
ENSMUSG00000076613	−1.53	1.60 × 10^−3^	Ighg2b
ENSMUSG00000076614	−1.53	1.20 × 10^−2^	Ighg1
ENSMUSG00000096715	−1.55	8.14 × 10^−3^	Igkv3-4
ENSMUSG00000096499	−1.57	1.20 × 10^−2^	Ighv1-5
ENSMUSG00000076594	−1.62	6.66 × 10^−3^	Igkv6-13
ENSMUSG00000094335	−1.62	1.19 × 10^−3^	Igkv1-117
ENSMUSG00000003379	−1.64	1.23 × 10^−3^	Cd79a
ENSMUSG00000094433	−1.75	1.23 × 10^−3^	Igkv5-43
ENSMUSG00000076563	−1.93	2.28 × 10^−4^	Igkv5-48
ENSMUSG00000095127	−2.01	7.43 × 10^−5^	Ighv1-82

**Table 2:** Genes and gene paralogs shared by the ILC2 WT and ILC2 KO at 10 dpi.

WT_CONTROL VS. WT_NB_10DPI				KO_CONTROL VS. KO_NB_10DPI		
Ensembl_ID	log2(FC)	Gene Name		Ensembl_ID	log2(FC)	Gene Name
ENSMUSG00000013653	4.25	1810065E05Rik	**Overlapping DEGs between WT and KO at 10 dpi**	ENSMUSG00000013653	−0.95	1810065E05Rik
ENSMUSG00000050296	2.54	Abca12		ENSMUSG00000050296	−0.98	Abca12
ENSMUSG00000095649	2.02	Gvin-ps3		ENSMUSG00000095649	−1.17	Gvin-ps3
**ENSMUSG00000071356**	**4.02**	**Reg3b**		**ENSMUSG00000071356**	**−0.99**	**Reg3b**
**ENSMUSG00000030017**	**3.34**	**Reg3g**		**ENSMUSG00000030017**	**−0.91**	**Reg3g**
ENSMUSG00000025993	2.2	Slc40a1		ENSMUSG00000025993	−1.41	Slc40a1
ENSMUSG00000043705	−3.82	Capn13	**Gene paralogs between WT and KO at 10 dpi**	ENSMUSG00000054083	0.91	Capn12
ENSMUSG00000069922	−2.42	Ces3a		ENSMUSG00000055730	0.94	Ces2a
ENSMUSG00000004267	−2.04	Eno2		ENSMUSG00000060600	0.79	Eno3
ENSMUSG00000013643	2.46	Lypd8		ENSMUSG00000026344	0.88	Lypd1
ENSMUSG00000037145	2.9	Lypd8l		ENSMUSG00000061068	−0.87	Mcpt4
ENSMUSG00000022227	−2.38	Mcpt1		ENSMUSG00000045725	0.79	Prr15
ENSMUSG00000022226	−2.4	Mcpt2		ENSMUSG00000051079	−0.94	Rgs13
ENSMUSG00000043795	−2.2	Prr33		ENSMUSG00000020641	0.77	Rsad2
**ENSMUSG00000079516**	**5.16**	**Reg3a**		ENSMUSG00000024818	0.97	Slc25a45
**ENSMUSG00000068341**	**2.47**	**Reg3d**		ENSMUSG00000070563	−0.96	Spaca4
ENSMUSG00000037627	−3.95	Rgs22		ENSMUSG00000027801	0.88	Tm4sf4
ENSMUSG00000038530	−2.5	Rgs4		ENSMUSG00000056133	1.27	Unc93a2
ENSMUSG00000039096	−2.88	Rsad1				
ENSMUSG00000031633	−2.02	Slc25a4				
ENSMUSG00000080316	−2.35	Spaca6				
ENSMUSG00000038623	−3.03	Tm6sf1				
ENSMUSG00000067049	−2.3	Unc93a				

**Table 3:** Over-enriched gene and pathway results for the wild-type response to *N. brasiliensis* at 10 dpi. Terms in bold consist of all upregulated genes; terms in italics are all downregulated for a given term.

Term Id	Term Description	# Genes	Enrichment Score	Genes
**GO:0061844**	**Antimicrobial humoral immune response mediated by antimicrobial peptide**	**14**	**6.63**	**Mmp7, Xcl1, Reg3g, Ang,** **Defa21, Defa24, Gm15308, Dmbt1, Reg3b, Defa-rs1, Ccl28, Reg3a, Cxcl9, Rpl39**
GO:0050830	Defense response to Gram-positive bacterium	14	5.59	Hck, Mmp7, Reg3g, Zg16, Hmgb2, Ang, Defa21, Defa24, Gm15308, Dmbt1, Lyz1, Reg3b, Defa-rs1, Rpl39
GO:0050829	Defense response to Gram-negative bacterium	14	5.19	Mmp7, Reg3g, Pycard, Serpine1, Nlrp10, Hmgb2, Defa21, Defa24, Gm15308, Dmbt1, Lyz1, Reg3b, Defa-rs1, Lypd8
CL:36134	Mixed, incl. defensin and protease inhibitors	17	6.04	Serpina1f, Ceacam10, Cercam, AY761184, Gm15315, Defa21, Gm14851, Gm10104, Defa24, Gm14850, Gm15308, Dmbt1, Def-rs1, Defa22, Gm15284, Lypd8, Gm15293
CL:25609	Cell wall disruption in other organisms, and Cobalamin (Cbl, vitamin B12) transport and metabolism	3	8.42	Reg3g, Reg3b, Reg3a
**CL:36334**	**Defensin/corticostatin family, and peptidyl-arginine ADP-ribosylation**	**7**	**6.04**	**Gm15315, Defa21, Gm14851, Defa24, Gm14850, Gm15284, Gm15293**
*CL:16259*	*Mixed, incl. macrophage proliferation and CD59 antigen, conserved site*	*4*	*4.87*	*Retnla, Mgl2, Chil3, Siglec5*
**CL:25564**	**Mixed, incl. activation of matrix metalloproteinases and cell wall disruption in other organisms**	**6**	**6.86**	**Reg3g, Klk1, Reg3d, Reg3b, Reg3a, Sycn**
**CL:25566**	**Mixed, incl. Cobalamin (Cbl, vitamin B12) transport and metabolism and cell wall disruption in other organisms**	**5**	**7.23**	**Reg3g, Reg3d, Reg3b,** **Reg3a, Sycn**
**MMU-1462054**	**Alpha-defensins**	**12**	**6.44**	**AY761184, Gm15315, Defa21, Gm14851, Gm10104, Defa24, Gm14850, Gm15308,** **Defa-rs1, Defa22, Gm15284, Gm15293**
MMU-6803157	Antimicrobial peptides	20	3.44	Prtn3, Clu, Reg3g, Rnase2a, AY761184, Ear2, Gm15315, Defa21, Gm14851, Gm10104, Defa24, Gm14850, Gm15308, Reg3d, Reg3b, Def-ars1, Defa22, Gm15284, Reg3a, Gm15293
MMU-211859	Biological oxidations	15	4.22	Mgst1, Ptgis, Cyp46a1, Sult1c, Cyp2c55, Maoa, Fmo3, Aadac, Aldh1b1, Cyp3a59, Nr1h4, Cyp3a25, Ugt2b5, Ces3a, Ugt2b36
*BTO:0001485*	Muscular system	27	2.47	Cnn1, Srpk3, Casq1, Ckm, Serpinb9b, Timp1, Slc2a4, Tpm2, Itgb1bp2, Slc25a4, Pdlim3, Tagln, Fn1, Gja1, Cav3, Sgcg, Dysf, Dio2, Trdn, Tnnc2,Sgca, Nppa, Hspb7, Myh7, Tnnt2, Dtna, Tnnt3
*BTO:0006111*	*M2 macrophage*	*3*	*6.44*	*Arg1, Retnla, Chil3*
**KW-0211**	**Defensin**	**14**	**6.35**	**Defb40, AY761184, Gm15315, Defa21, Gm14851, Gm10104, Defa24, Gm14850, Gm15308, Defa-rs1, Defa22, Gm15284, Gm15293, Defa2**
KW-0929	Antimicrobial	22	5.20	Irg1, Reg3g, Defb40, AY761184, Gm15315, Defa21, Gm14851, Gm10104, Defa24, Chil1, Gm14850, Gm15308, Lyz1, Reg3b, Def-ars1, Defa22, Gm15284, Reg3a, Slpi, Gm15293, Defa2, Vgf

## Data Availability

The data are available in SRA accession # PRJNA1219174.

## Supplementary Materials

The following supporting information can be downloaded at https://www.mdpi.com/article/10.3390/pathogens14060571/s1: Table S1: WT_control vs. WT_NB.

## Abbreviations

The following abbreviations are used in this manuscript:

DPI	days post-infection
NB	*Nippostrongylus brasiliensis*
ILC	innate lymphoid cell
